# Energy expenditure and body composition in a hibernator, the alpine marmot

**DOI:** 10.1007/s00360-022-01466-1

**Published:** 2022-11-06

**Authors:** Thomas Ruf, M. Michel, F. Frey-Roos, S. Flatz, F. Tataruch

**Affiliations:** 1 Department of Interdisciplinary Life Sciences, Institute of Wildlife Ecology, University of Veterinary Medicine, Vienna, Savoyenstr. 1, 1160 Vienna, Austria; 2 Present Address: Institute of Wildlife Biology and Game Management, University of Natural Resources and Life Sciences, Vienna, Gregor Mendel-Str. 33, 1180 Vienna, Austria

**Keywords:** Basal metabolic rate, Gastrointestinal tract, Fat reserves, Climate

## Abstract

Visceral organs and tissues of 89 free-living alpine marmots (*Marmota marmota*) shot during a population control program in Switzerland, were collected. Between emergence from hibernation in April to July, the gastrointestinal tract (stomach to colon) gained 51% of mass and the liver mass increased by 24%. At the same time, the basal metabolic rate (BMR), determined with a portable oxygen analyzer, increased by 18%. The organ masses of the digestive system (stomach, small intestine, caecum, large intestine) were all significantly correlated with BMR. Interestingly, the mass of abdominal white adipose tissue (WAT) and of the remaining carcass (mainly skin and bones) were also significantly correlated with BMR. These results indicate that the gastrointestinal tract and organs involved in digestive function are metabolically expensive. They also show that it is costly to maintain even tissues with low metabolic rate such as WAT, especially if they are large. Heart and kidneys and especially brain and lungs did not explain a large proportion of the variance in BMR. Marmots increased the uptake of fat prior to hibernation, both by selective feeding and enhanced gastrointestinal capacity. Large fat reserves enable marmots to hibernate without food intake and to reproduce in spring, but at the cost of an elevated BMR. We predict that climate changes that disturb energy accumulation in summer, increase energy expenditure in winter, or delay the emergence from hibernation in spring, such as the occurrence of storms with increasing frequency, will increase mortality in alpine marmots.

## Introduction

Alpine marmots prefer relatively cool areas in alpine regions (Arnold 1999). Apparently, their body shape and insulation are optimal for activity in late winter/early spring. On hot days in summer, they even quit forging for plants and stay in their cool underground burrows at noon (Türk and Arnold 1988).

Marmots are forced to reproduce early in the year, while their territory is still covered by a 2–3 m snow layer. This is to give the young enough time to grow and fatten prior to their first winter. Late in fall, marmots mostly escape from harsh conditions by hibernating for 6–7 months. During deep torpor in hibernation, marmots as most hibernators drastically lower their metabolic rate and energy expenditure by ~ 95% (Ortmann and Heldmaier 2000).Like almost all hibernators, they interrupt hibernation for periodic arousals which seem to be driven by the need to restore an accumulating metabolic imbalance (Ruf et al. 2022). In the case of marmots, this repeated rewarming occurs on average every 12 days (Arnold 1999).

Alpine marmots belong to the fat-storing hibernators that cease to forage prior to hibernation in fall (Dark 2005). This is true for a majority of species, while fewer hibernators continue to take up food over winter. During hibernation, marmots live on body energy stores alone, i.e., for more than half a year. However, some hibernators rely on body energy reserves for a full year or even somewhat longer (Geiser 2007; Hoelzl et al. 2015).

Alpine marmots support replenishment of their body energy reserves by increasing the size of the gastrointestinal tract, starting only a few days after emergence (Hume et al. 2002), and then increase their food intake. Stomach, small intestine, caecum, and colon all increase in length and at least double their mass over the active season. Maximum size was reached in July and organs often shrank again towards September (Hume et al. 2002). Mucosal thickness maxima of the duodenum, ileum, caecum, and proximal colon were observed during late summer. Mitotic activity in the duodenum and ileum increased significantly during maximum growth (Hume et al. 2002).

Here, we investigate if, and to which degree, these costs for the machinery to build up fat depots involve extra energy expenditure. It has been argued that alimentary organs only play a minor role in determining basal metabolic rate (BMR), whereas energy turnover would be dominated by organs like the heart and kidneys (Daan et al. 1990). Therefore, it was even postulated that the relation between BMR and the size of the heart seems to be a general phenomenon in mammals and birds (Meerlo et al. 1997). However, more recent studies point to an important role of alimentary organs, namely the small intestine (Konarzewski and Książek 2013; Książek et al. 2004). Also, a previous study on alpine marmots has shown that the growth of the alimentary tract is immense, and the small intestine growth is among the largest recorded for a mammal (Hume et al. 2002). We aimed to see whether in marmots the gastrointestinal tract is indeed one of the most metabolically intense vertebrate organs and costly to maintain (Stevens and Hume 2004).

BMR was measured in the present study using a portable oxygen analyzer. By this, we avoided lengthy travels of animals to the laboratory and feeding them artificial food for maintenance. Both may pose a stress on the animals and this could affect their metabolism. Further, we aimed to not merely investigate the role of the gut, but study the contribution of all visceral organs to BMR.

However, a seasonal switch in food uptake seems particularly important as it creates a potential conflict. On one hand, hibernators must gain large body fat reserves to sustain maintenance for several months. This requires a digestive system that is large and efficient enough to acquire a surplus of energy. On the other hand, large alimentary organs may create an elevated BMR, which counteracts possible energy savings, at least in normothermic animals. In the present study, we investigate if and how much alimentary and other organs contribute to energy expenditure in alpine marmots. We hypothesized that marmots would sustain large digestive organs only in summer and fall, when they gain energy reserves. This temporal limitation should result in large changes of the size and mass of the alimentary tract, and result in corresponding changes of BMR.

Due to these constraints, one could argue that hibernators should only incorporate just sufficient energy to survive a future shortage. However, large fat stores are also used for an early hibernation onset and to minimize the time spent a very low body temperature during hibernation (Bieber et al. 2014; Hoelzl et al. 2015; Zervanos et al. 2014). Hence, it has been suggested that hibernation has not only advantages but also risks and drawbacks (Humphries et al. 2003). However, the cost of having large body fat reserves and an initially high body mass is still much smaller than the benefits of hibernation in terms of energy savings.

## Methods

### Animals and capture

Alpine marmots are large (up to 6 kg), diurnal, herbivorous, hibernating rodents that live in mountains above 800 m asl. They live in family groups and reproduce once a year soon after emergence from hibernation. Young are born after 34 days of pregnancy and appear above ground in early July (Arnold 1990).

Adult marmots from an area close to Avers, Grisons, Switzerland were captured with live traps (Tomahawk, Wisconsin USA) that were placed into burrow entrances. The mean day of emergence from hibernation was April 15. Females in spring had often enlarged nipples. However, we could not determine with any certainty that these were due to a current or a previous lactation. After capturing, each animal was weighed (Pesola, Basel, Switzerland), sexed, marked with Nyanzol fur dye (Nyanzol, Belmar, Missouri, USA) and placed into a wooden box (25 × 25 × 47 cm; thickness 0.5 cm) with steel grids as front and back cover. Subsequently the box was kept in a sheltered place over night, and the animals were maintained without food to ensure that the measurements of BMR were made on the next day (after > 12 h) in the post-absorptive state.

Following BMR measurement, rectal temperature was measured (ALMEMO, Ahlborn, Holzkirchen, Germany) and 2–4 ml of blood was taken for another parallel study. Marmots were then released into their home territory close to the site of capture. Animals were shot 48–120 h after their release by professional hunters employed by the canton of Grisons, if possible, by a shot in the neck to spare any organs. Hunting was carried out because marmots and their burrows are a nuisance to farmers and cattle, and marmots are managed by the canton.

### Measurements of BMR

BMR was measured using a portable O_2_ analyzer based on gas analyzer with chemo-electrical sensors (Gasmonitor O_2_-25, Bieler & Lang, Achern, Germany; accuracy < 0.02 vol%), very similar to the devices used before for measuring O_2_ consumption of animals in tree holes or nest boxes (e.g., Schmid et al. 2000; Dausmann et al. 2009). An airtight plastic box (26 × 34 × 50 cm) served as a respirometry chamber. The marmot in its cage, that had only steel grids as front and back covers, was placed into the chamber. The empty respirometry chamber had a volume of 44.2 l. Ambient air was drawn through the respirometry chamber using a car ventilation pump based on a radial fan (Metzger, Vienna, Austria).

Flow rates of dry air were continuously measured by a mass flow meter (AWM5104V, Honeywell, Freeport, Illinois, USA). The system was battery powered over several hours. Therefore, a voltage stabilizer (LM317T) was used to maintain the flow meter supply voltage constantly at + 9.0 VDC. Although this arrangement significantly reduced problems due to electronic drift, a 2/3-way solenoid valve (Type 305-C-03, O-B-MS, impulse-switched, Buerkert, Ingelfingen, Germany) was additionally installed. This valve allowed ambient reference air to be pumped through sensor cells temporarily to enable zero-checks at regular intervals. The O_2_ sensor signal was recorded by a laptop computer (Contura Aero 4/25, Compaq) using a 16-bit A/D converter (DACpad-71B, Advantech, connected via the PCMCIA slot of the laptop). At 5 min intervals, the recording software switched the solenoid valve to supply the O_2_ sensors with reference air for 1 min (while maintaining a steady flow of air to the animal in the respirometry chamber).

To allow measurements at a defined temperature inside the thermoneutral zone of marmots (TNZ) (Ortmann and Heldmaier 2000), the respirometry chamber could be heated to about 24 °C. A 450 W power resistor, built into an aluminum tube, with an added fan was used as a heating element. A thermistor attached to a digital thermometer (ALMEMO 2190-2, accuracy ± 0.25 °C, Ahlborn, Holzkirchen, Germany) was used to continuously measure the temperature inside the respirometry chamber. At all temperatures, the air in the respirometry chamber was raised from ambient temperature (minimum 3 °C) to 24 ± 0.5 °C within 2 min or less. Due to the control circuit, temperature inside the chamber never varied more than ± 0.5 °C, irrespective of changes in the outside temperature.

The O_2_ analysis unit was calibrated before each measurement. First ambient reference air was directed over O_2_ sensors. The output signals of the cells were adjusted to produce a defined reference signal (+ 0.5 VDC) corresponding to zero O_2_ difference, i.e., an O_2_ content of 20.95 Vol%. Second, a commercially available calibration gas with an O_2_ content of 20.017 Vol%. (Linde GAS, Stadl-Paura, Austria, brought to the study site in a 10 l bottle) was directed over the sample cell. The exact O_2_ content of this gas had been determined under laboratory conditions to the nearest 0.005 Vol% with an Oxor 610 (Maihak, Hamburg, Germany) O_2_ analyzer. As the response of the O_2_ sensor system was known to be completely linear (Schmid et al. 2000), this two-point calibration was fully sufficient.

### O_2_ consumption analysis

Depending on the mass of the animal, the airflow was adjusted between 500 and 700 l h^−1^. A self-written program in QuickBasic 4.5 running on the laptop monitored all incoming signals at 1 min intervals and stored flow rate, oxygen consumption, and the temperature in the respirometry chamber. O_2_ consumption under STPD conditions was calculated as described in Lighton (2008). Since the volume of the respirometry chamber was large compared with the average air flow rate, there was some damping of actual changes in O_2_ consumption, Therefore, we calculated “instantaneous” rates of O_2_ consumption using Eq. (3) of Bartholomew et al. (1981) (see also Lighton 2008). To determine BMR, we computed the mean of the two lowest measurements of O_2_ consumption within each recording session. BMR is given as ml O_2_ h^−1^. Each recording of a marmot lasted approximately for 3 h.

### Tissue collection

After determining their mass (GP 3100 S-G Sartorius, Göttingen, Germany), carcasses were opened and we removed stomach, small intestine, caecum, large intestine, liver, kidneys, spleen, heart, lungs, brain, eyes, gonads, and adrenals. In some cases, certain organs were destroyed by the bullets and discarded. Used sample sizes are given in Table 1. We also determined the mass of abdominal white adipose tissue (WAT) and of the remaining parts (mainly bone and skin but including subcutaneous fat; measured to the nearest 5 g using a spring balance; Pesola, Basel, Switzerland). The different sections of the gastrointestinal tract were squeezed empty and washed out with physiological saline solution. Organs and tissues were then blotted and weighed.

**Table 1:** Organ or tissue mass at different seasons

Tissue	*N*	EDF	*F*	*P*	April 15	July 1	Sep. 15
Stomach	89	1.73	36.10	< 0.0001	23.7	33.6	39.9
Small intestine	89	3.03	30.76	< 0.0001	33.9	95.4	87.3
Caecum	89	2.71	39.33	< 0.0001	20.6	35.4	37.6
Large intestine	89	1.90	15.40	< 0.0001	27.9	35.7	38.9
Kidneys	89	1.53	13.49	< 0.0001	13.6	16.4	18.0
Liver	89	1.65	15.78	< 0.0001	72.5	91.9	102.1
Gallbladder	89	2.78	6.36	0.0008	3.1	7.7	5.2
Heart	89	1.00	1.12	0.2930	13.0	13.4	13.7
Lungs	87	1.00	0.98	0.3260	23.0	24.6	26.1
Brain	69	1.00	2.42	0.1240	12.5	12.9	13.4
Eyes	81	2.04	1.80	0.1930	4.0	3.8	4.0
Spleen	89	1.00	2.28	0.1350	2.1	2.2	2.3
Adrenals	84	1.08	22.26	< 0.0001	0.2	0.3	0.3
Testes	63	1.00	35.99	< 0.0001	4.3	2.6	0.9
Ovars and uterus	26	1.00	4.36	0.0476	41.5	16.5	≤ 0
WAT	89	3.19	20.52	< 0.0001	182.2	86.9	299.5
Bones and skin	89	1.84	25.63	< 0.0001	2279	2541	3079
Body mass	89	1.55	34.13	< 0.0001	2919	3477	4258

Results from general additive models. EDF: degrees of freedom (wiggliness) of the spline term; *F* and *P* values. The three columns on the right are model predictions for the end of hibernation (April 15), midsummer (July 1), and prior to hibernation onset (Sept 15)

For logistic reasons, we could no not determine dry weights of all organs. Dry masses of organs and tissues that could be dried at 60 °C (stomach, small intestine, large intestine, caecum, liver, kidneys, heart, lungs, brain, spleen, and WAT) were closely correlated with fresh masses (*r* = 0.924–0.999; *n* = 27–60;) except for moderate correlations among adrenals (*r* = 0.76; *n* = 22) and eyes (*r* = 0.80; *n* = 26). Since fresh masses are most relevant for energy budgets (see “Results” and “Discussion”), they are provided throughout. In the course of three years (2002–2004), we obtained body composition and BMR simultaneously of 89 marmots.

### Organ chemistry

Dried subsamples were ground in a mortar. Sample sizes varied and are given in Table 2. Nitrogen was determined according to the Kjeldahl method (Lang 1958). Crude fat contents were determined using the Soxhlet method (Soxhlet 1879) after extraction in petrolic ether for 5 h, followed by drying and weighing of the sample. Ash content was determined after burning 0.5 g of the sample at 550 °C overnight. Carbohydrates were determined by subtracting the protein, fat, and ash content from the sample material mass, using a nitrogen-to-protein conversion factor of 6.25. The determination of tissue chemical composition was carried out twofold.

**Table 2:** Mean crude fat content (%) of major organs in marmots in spring, summer, and fall

Tissue	*N*	Spring Day 113–150	Summer Day 188–202	Autumn Day 245–260
Stomach	27	5.05	5.75	6.93
Small intestine	51	9.82	8.60	9.02
Caecum	27	5.33	6.12	9.33
Large intestine	27	5.08	6.96	6.34
Kidneys	60	7.59	8.96	8.60
Liver	60	10.11	7.52	7.18
Heart	27	4.79	6.02	6.96
Lungs	27	10.77	11.07	11.49
Brain	24	47.23	46.37	47.22
Eyes	19	28.25	22.14	22.73
Spleen	27	5.73	7.58	7.81

### Statistical analysis

Data were analyzed in R (R Core Team 2022). Residuals of models from both BMR and organ masses were approximately normally distributed as determined by histogram and quantile–quantile plots. In all models for BMR comparison, total body mass was entered as a covariate. This avoids the error inherent in dividing O_2_ consumption by mass (Fernández-Verdejo et al. 2019). Since most variables changed in a non-linear way, we computed general additive models using the default TP-splines for all effects (package mgcv; Wood 2011). We provide EDF, the estimated degrees of freedom that indicate the ’wiggliness’ of a smooth term (1 = linear) as well as *F* and *P* values of tests for their equality to zero. Two means were compared with *t* tests using Welch’s modification to the degrees of freedom for unequal variances. Correlations between variables were given as Pearson’s product moment coefficients.

## Results

**Fig. 1:**
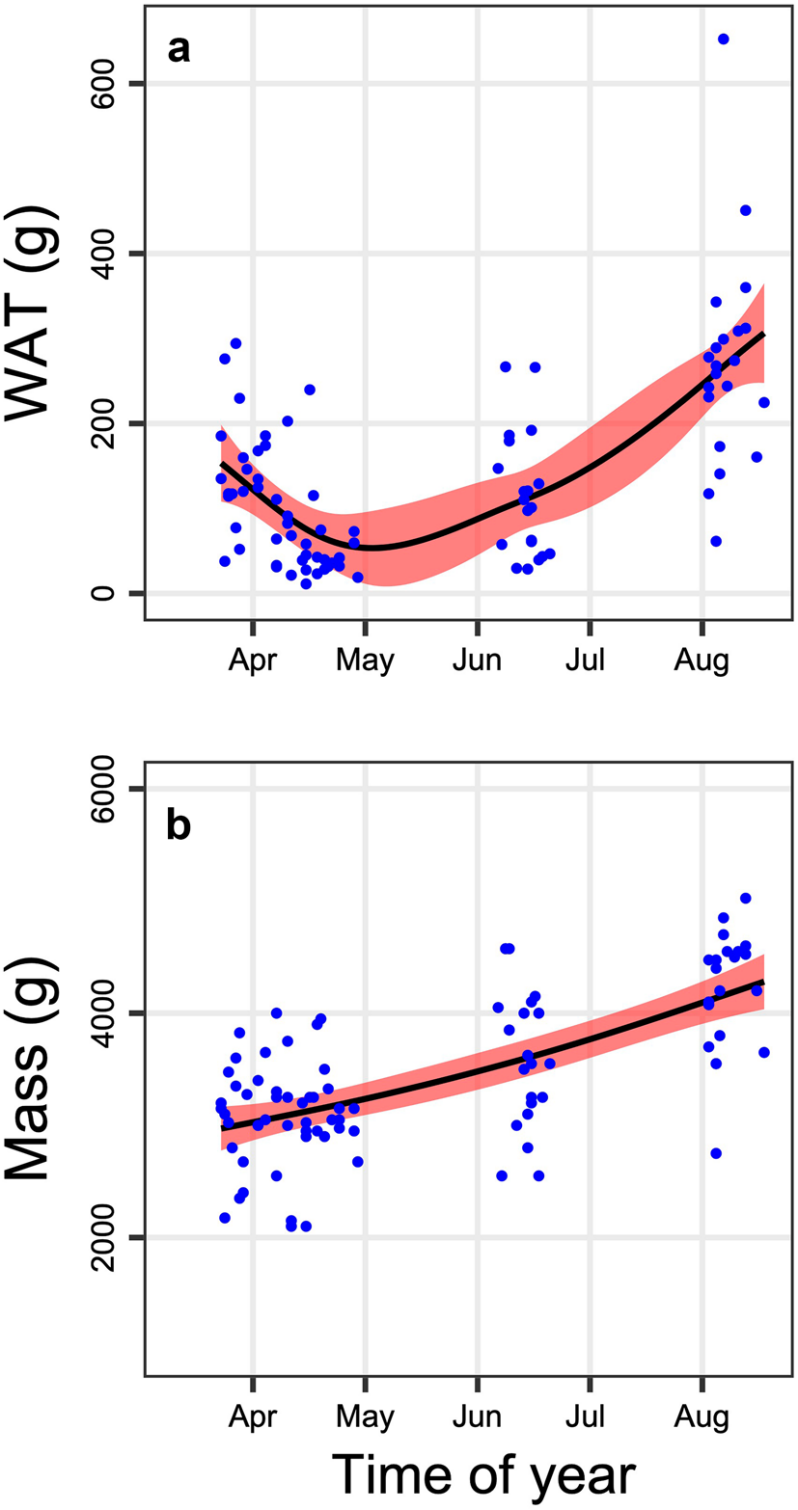
**a** Seasonal time course of abdominal WAT and of **b** total body mass in alpine marmots. General additive models with 95% confidence intervals (red) and residuals (blue)

The abdominal white adipose tissue was first depleted in late spring and then gained mass (Fig. 1a). Marmots increased mass substantially by increasing remaining tissues, mainly abdominal WAT and subcutaneous fat (Fig. 1; Table 1; subcutaneous fat is part of “bones and skin”). The incorporation of fat into organs as the season progressed was minimal (Table 2). The stomach matter fat content significantly increased with season (*n* = 27; EDF = 1.96, *F* = 7.60, *P* < 0.0001). It was 7.82 ± 0.87% fat content in fall vs. 4.19 ± 0.36% fat content in spring/summer.

**Fig. 2:**
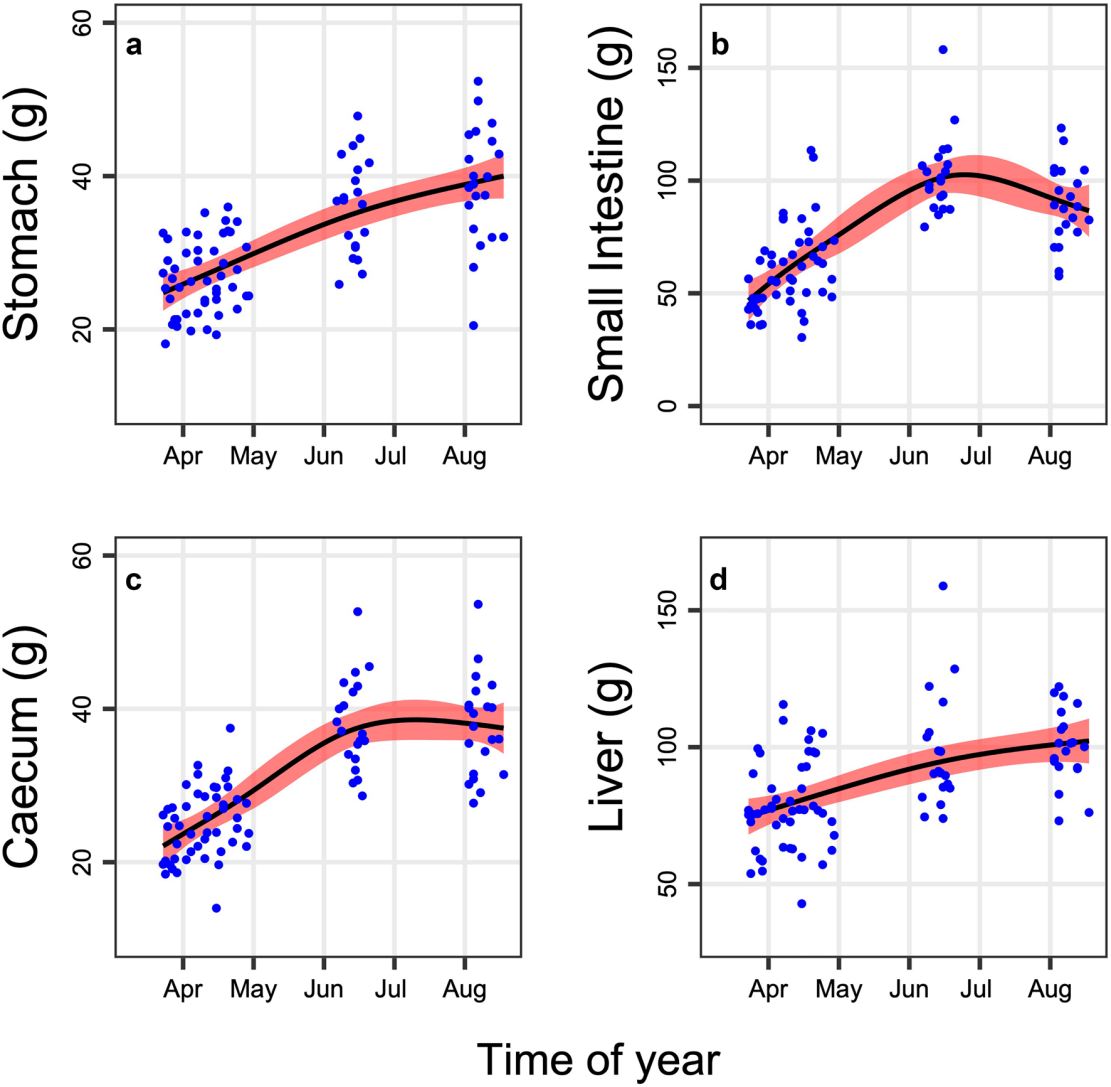
Seasonal time course of **a** stomach, **b** small intestine, **c** caecum, and **d** liver mass in alpine marmots

The empty gastrointestinal tract and liver in marmots increased significantly between emergence from hibernation in spring, and immergence in fall (Fig. 2; Table 1). In sum, the gastrointestinal tract gained 151% between spring and summer. The empty stomach alone made less than 1% of the body mass, but changes in stomach mass explained 53% of the variance in total mass. Other organs such as heart, lungs, and brain increased only very little or not at all (Table 1). Interestingly, the small intestine and the caecum peaked already in summer, not only immediately prior to hibernation (Fig. 2).

**Fig. 3:**
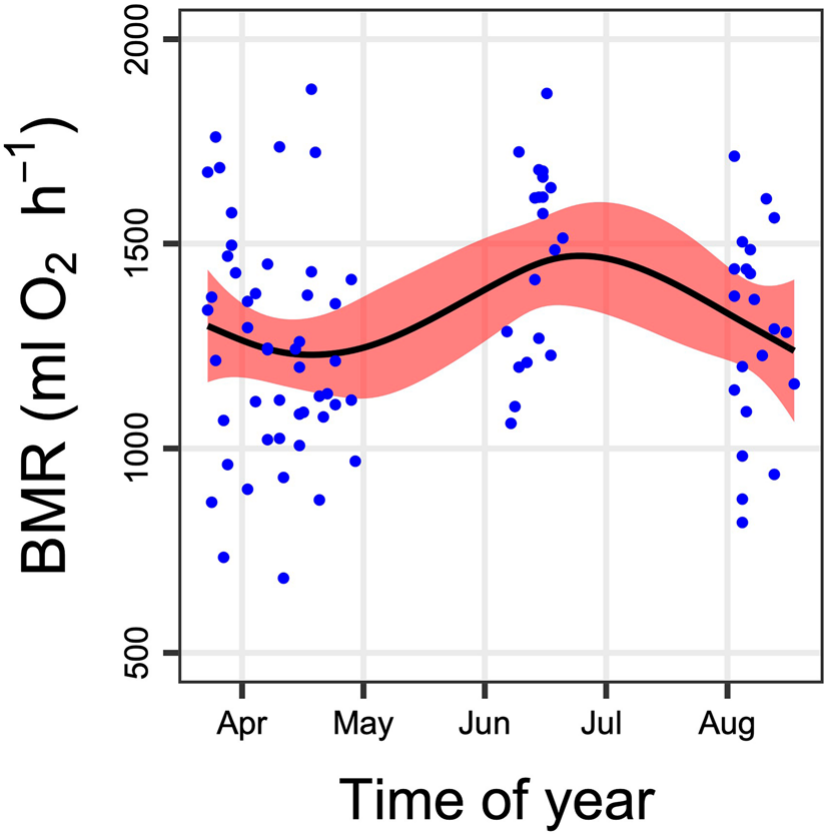
Seasonal time course in basal metabolic rate of alpine marmots

**Fig. 4:**
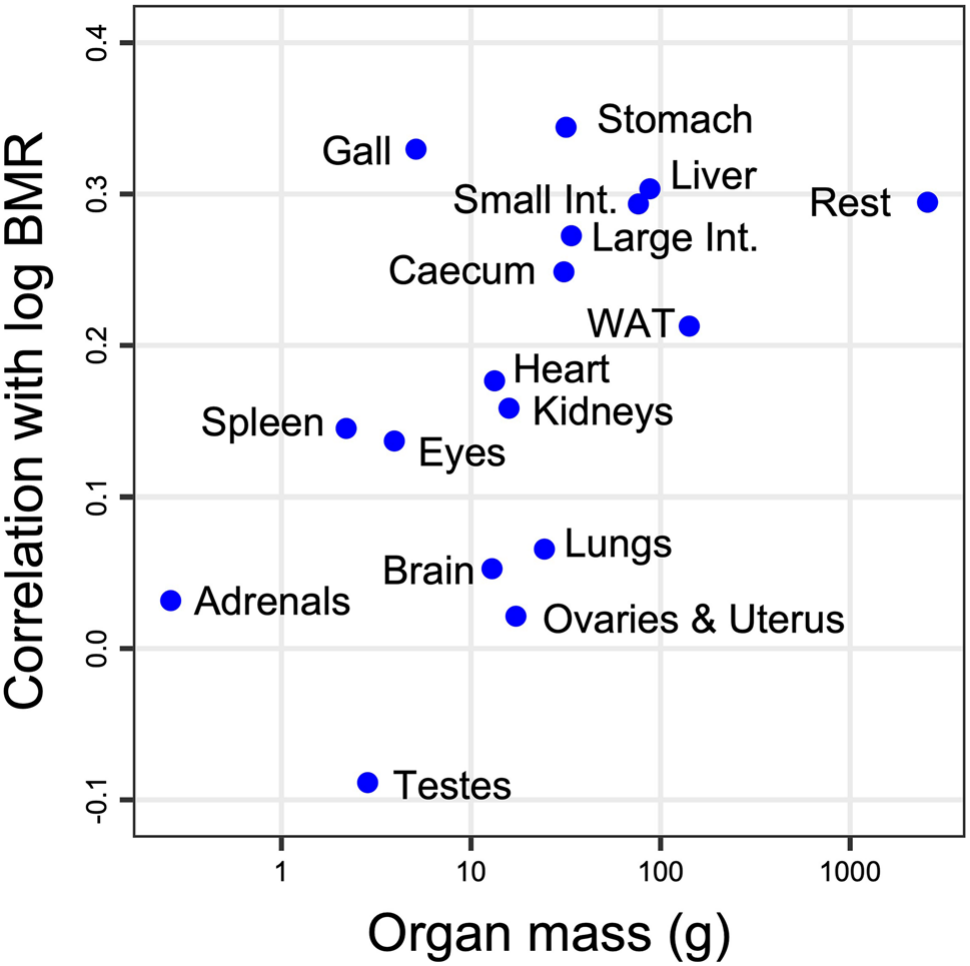
Correlation between mean organ/tissue mass and BMR (log10 transformations of both variables). All correlation coefficients above 0.2 were significant (*P* < 0.05). Some organs (e.g., stomach) contributed more to BMR than predicted by their mass. Only the relation between testes size and BMR was negative. Int. means intestine

BMR also peaked in midsummer and was slightly lower in spring and fall (EDF = 3.00, *F* = 3.56, *P* = 0.0279, Fig. 3). BMR was a slightly curvilinear function of body mass, even when both variables were log transformed (EDF = 3.425, *F* = 4.30, *P* < 0.0001), and was correlated with the size and function of major organs/tissues (Fig. 4). The mass of the stomach and all organs of the digestive system (intestine, liver) significantly correlated with log(BMR) (Fig. 4). Interestingly, the size of the abdominal WAT and of remaining tissues (mainly skin and bone) also correlated with metabolism. Certain organs, such as the stomach, contributed to BMR more than expected by their mass alone. Others, like the brain, played almost no role in determining BMR (Fig. 4).

Mass-corrected BMR in spring and summer, despite reproduction, was slightly lower in females and not significantly different from males (*t* = 0.390; *P* = 0.698). Also, rectal temperature played no role for the level of BMR (*t* = 0.164; *P* = 0.10).

## Discussion

The yearly cycle of fat gain and loss is typical for hibernators relying on body energy stores and particularly for sciurids, such as the alpine marmot (Dark 2005). Marmots and ground squirrels typically emerge from hibernation with﻿ substantial body fat reserves that are used up during reproduction, which seems common in large hibernators (Huang and Morton 1976; Morton 1975). The later the marmots were trapped in April, the lower were their fat reserves (Fig. 1). However, WAT increased again in the second half of the year, and adults in the present study increased body mass from spring to fall by 68%, which is quite typical (Dark 2005).

Fattening in hibernators usually involves hyperphagia, and the orexigenic hormone ghrelin reaches peak values during the fattening period in fall (Florant and Healy 2011). Besides ghrelin, changes in circulating leptin and insulin, as well as in nutrients (glucose, and free fatty acids), and cellular enzymes such as AMP-activated protein kinase (AMPK) determine the activity of neurons involved in the food intake pathway. Importantly, during the fattening phase, hibernators become temporarily insensitive towards leptin, the hormone that normally signals white fat content and limits lipid uptake and deposition. In hibernators, leptin can be temporarily disassociated from adiposity (review in Jastroch et al. 2016). As outlined in Dark (2005), this seasonal change in lipid content is programmed and part of the circannual cycle. For example, ground squirrels quickly recover from the surgical excision of a substantial amount of WAT (Dark et al. 1984), and temporary food restriction does not prevent projected increases in body mass (Barnes and Mrosovsky 1974). It seems unlikely that the yearly cycle in fattening and fat loss is governed by photoperiod. As outlined by Davis (1976), various lengths of photoperiod or changes of photoperiod throughout the year have failed to cause animals to enter torpor or to undergo the seasonal changes typical for hibernators. There are only a few exceptions of hibernators in which photoperiod seems to play a role (e.g., Darrow et al. 1986). Many hibernators kept in constant conditions showed a cycle of about 11 months (Pengelley and Fisher 1961). Thus, the consumption of food apparently follows an annual rhythm (Davis 1976). This circannual rhythm may involve the expression of deiodinase (DIO2) in the tanycytes adjacent to the third ventricle of the brain. As reviewed by Jastroch et al. (2016), there is evidence to suggest that tanycytes mediate seasonal responses in food intake and body weight. These cells are able to receive metabolic information from the cerebrospinal fluid and the blood, and have been identified as major players in the seasonal control of energetic states in mammal and apparently have an important role in seasonal anorexia (Junkins et al. 2022; Bolborea and Langlet 2021).

Interestingly, the fat deposition phase in fall (Fig. 1) was accompanied by an increased lipid content of the stomach matter. The crude fat content in the food plants of herbivorous mammals is typically less than 3.5% (review in Palmquist and Jenkins 1980). Thus, the only way to enrich the stomach content of marmots to 4.2% in spring, which is already high, to a full 7.8% lipids in fall, is by the selective uptake of fatty plants. Other marmot species are known to selectively ingest certain plants, and some herbivores clearly prefer fats (Schai-Braun et al. 2015; Stallman and Holmes 2002). As alpine marmots are able to retain n-6 polyunsaturated fatty acids that improve hibernation (Bruns et al. 2000; Arnold et al. 2011), it seems likely that they also should preferentially take up fats in fall.

Large increases in food uptake were aided by the seasonal growth of digestive organs such as stomach, intestine, and liver. A profound increase in the organ mass of alpine marmots, with large changes especially in the small intestine, (+ 281%, Table 1) was reported before, and had been associated with large increases in the mitotic index (Hume et al. 2002). Similar extents at even greater rates are only known from ectothermal snakes after feeding (Secor and Diamond 1995, 2000). Interestingly, food uptake in these ‘sit and wait predators’ can be just as infrequent as in hibernators and has similar consequences (Secor and Carey 2016). In marmots, increases in size and activity of the gastrointestinal tract appear to be a response to ingested food rather than to an endogenous signal (Hume et al. 2002).

In the present study, alpine marmots mainly increased stomach, small intestine, caecum, and colon over the active season, as was reported before (Hume et al. 2002). In addition, there was a significant mass increase in the liver. This growth of the digestive system was paralleled by an increase in BMR that peaked in midsummer (Figs. 2, 3). Hence, it is more than likely that gut and liver increasingly contributed to BMR. The masses of these organs were clearly correlated with BMR, highlighting the cost of maintaining such an active digestive system. Interestingly, the large abdominal WAT, although it has a low metabolic rate according to common view (Benedict 1915; Daan et al. 1990; Elia 1992), as well as the remainder of the carcass (mainly bone and skin) were also significantly correlated with BMR (Fig. 4). This relationship indicates that correlations act both ways and that high rates of BMR are required to heat up tissues well above the ambient temperature (a gradient that increases below the TNZ), even those tissues that are thought to have low metabolic rates.

The contribution of heart and kidneys was only moderate, and these organs did not change much in mass. Remarkably, the correlation of brain size with BMR was negligible. This contradicts, at least for marmots, that the brain is an expensive organ (Niven and Laughlin 2008; e.g., Heldstab et al. 2018). It also may mean that brain mass relative to body mass (Heldstab et al. 2018) is a questionable measure in fat-gaining hibernators. In marmots, for example, the relative brain mass decreased from 0.43% in spring to 0.32% in fall, which is a full third. Even if it is small, the brain should have significantly correlated with BMR if it was very costly.

Given their fat and body mass gain, alpine marmots have much higher total BMR in fall than in spring. Ortmann and Heldmaier (2000) already noticed that the mass-specific metabolic rate of heavy marmots in winter was much smaller than that of summer-active animals. This increase over summer in the energy expenditure of normothermic, non-hibernating animals is thus a severe trade-off inherent in preparation for the winter fast. Alpine marmots will have a higher energy expenditure when they start to hibernate (including during arousals) than when fat stores are almost depleted. Notably, the winter fast extents much after hibernation into the spring, the reproductive phase. As mentioned by Dark (2005), there are numerous reports of the dire consequences visited by late-season storms that delay emergence from hibernation, or of reduced food availability in the summer, which can reduce fat deposition. We predict that these events as a consequence of climate change may well lead to increased mortality in alpine marmots.

Importantly, changes in food availability pose a problem if, and only if, they are unpredictable. Differing from Norin and Metcalfe (2019), we think harsh environmental conditions alone can be overcome by controlled reductions of metabolic rate in hibernation or estivation. Only extreme, sudden environmental events, such as fires or storm, require immediate responses, e.g., daily torpor (Nowack et al. 2015; Stawski et al. 2017). After all, a high responsiveness to short-term conditions rather than predictable seasonal changes is a characteristic that separates daily torpor from hibernation (Ruf and Geiser 2015). In general, as a consequence of global climate change, we would expect to see a decline of fat-storing hibernators and an increase in populations of species capable of daily torpor.

## Data Availability

Data are available from the University of Vienna PHAIDRA data repository.
